# Intracerebral hemorrhage: who gets tested for methamphetamine use and why might it matter?

**DOI:** 10.1186/s12883-020-01967-y

**Published:** 2020-10-27

**Authors:** Sahar Osman, Zhu Zhu, Mark Farag, Leonid Groysman, Cyrus Dastur, Yama Akbari, Sara Stern-Nezer, Dana Stradling, Wengui Yu

**Affiliations:** Department of Neurology, University of California, Irvine, CA USA

**Keywords:** Intracerebral hemorrhage, Methamphetamine, Urine drug screen, Stroke prevention

## Abstract

**Background:**

Methamphetamine use is an emerging risk factor for intracerebral hemorrhage (ICH). The aim of this study was to investigate the use of urine drug screen (UDS) for identifying methamphetamine-associated ICH.

**Methods:**

This is a retrospective, single-center study of consecutive patients hospitalized with spontaneous ICH from January 2013 to December 2017. Patients were divided into groups based on presence of UDS. The characteristics of patients with and without UDS were compared. Factors associated with getting UDS were explored using multivariable analyses.

**Results:**

Five hundred ninety-six patients with ICH were included. UDS was performed in 357 (60%), and positive for methamphetamine in 44 (12.3%). In contrast, only 19 of the 357 patients (5.3%) had a documented history of methamphetamine use. Multivariable analysis demonstrated that patients screened with UDS were more likely to be younger than 45 (OR, 2.24; 95% CI, 0.26–0.78; *p* = 0.004), male (OR, 1.65; 95% CI, 0.44–0.84; *p* = 0.003), smokers (OR, 1.74; 95% CI, 1.09–2.77; *p* <  0.001), with history of methamphetamine use (OR, 10.48; 95% CI, 2.48–44.34; *p* <  0.001), without diabetes (OR 1.47; 95% CI, 0.471–0.975; *p* = 0.036), not on anticoagulant (OR, 2.20; 95% CI, 0.26–0.78; *p* = 0.004), with National Institutes of Health Stroke Scale (NIHSS) > 4 (OR, 1.92; 95%CI, 1.34–2.75; *p* <  0.001), or require external ventricular drain (EVD) (OR, 1.63; 95%CI, 1.07–2.47; *p* = 0.021. There was no significant difference in race (*p* = 0.319). Reported history of methamphetamine use was the strongest predictor of obtaining a UDS (OR,10.48). Five percent of patients without UDS admitted history of use.

**Conclusion:**

UDS identified 12.3% of ICH patients with methamphetamine use as compared to 5.3% per documented history of drug use. There was no racial bias in ordering UDS. However, it was more often ordered in younger, male, smokers, with history of methamphetamine use, without diabetes or anticoagulant use.

## Background

Methamphetamine use is increasing globally, having reached epidemic proportions in the West, and emerging as a significant public health issue [1, 2]. Its use is associated with cerebrovascular toxicity, both directly via damage to the endothelial lining and indirectly through potent sympathomimetic activity [3, 4]. Both chronic and acute use may lead to intracerebral hemorrhage (ICH), with previous studies reporting these patients faring worse [4–6]. Our previous study, looking at outcomes between methamphetamine and non-methamphetamine associated ICH, revealed no difference in ICH severity, length of stay (LOS), and functional outcome, which may be attributed to dedicated care in a neurological intensive care unit (ICU), something not available at all hospitals [7]. The only treatment for methamphetamine addiction is behavioral therapy [2, 8], highlighting the importance of early interventions to mitigate this potentially preventable, increasingly recognized risk factor for ICH [5–7, 9–11]. Methamphetamine is the most commonly abused drug by patients in treatment facilities in Orange County, totaling 44% of patients seeking substance abuse treatment [12].

It is critical to identify the use of methamphetamine in patients presenting with spontaneous ICH in order to understand the extent of this issue and better target resources for prevention. This may be particularly challenging, because patients may not provide history of use due to aphasia, depressed mental status, or unwillingness to admit use. Ancillary testing can be essential. At our comprehensive stroke center in Orange County, California, we use the urine drug screen (UDS) to identify methamphetamine use as a risk factor. The decision to order the test, however, is at the discretion of treating physicians which typically include the stroke team and emergency physicians.

The aim of this study was to investigate the use of UDS for identifying methamphetamine-associated ICH. We also examined the factors associated with the ordering of UDS and what proportion of patients with ICH at our facility are associated with methamphetamine use per UDS versus per documented history of methamphetamine use.

## Methods

### Study design and settings

This is a single-center, retrospective study of consecutive patients hospitalized with spontaneous ICH from January 2013 to December 2017 at the University of California, Irvine Medical Center (UCIMC) in Orange, California.

### Selection of participants

The prospectively maintained stroke center data for American Heart Association (AHA)-Get With The Guidelines-Stroke Registry was used to identify all the patients admitted with spontaneous ICH during the study period. Patient demographics and clinical data, including age, sex, race, past medical history, social history, home medications, heart rates and blood pressures (BP) within 24 h of admission, initial Glasgow Coma Scale (GCS) score, National Institutes of Health Stroke Scale (NIHSS) score, ICH locations, intraventricular hemorrhage (IVH), ICH score, UDS, neurosurgical interventions, and intubation in the ermergency department (ED), were abstracted from the electronic medical record.

Data abstractors consisted of two physicians trained in neurology. Charts were divided between the two with 20 patient overlap. One experienced physician trained the other on how to look for pre-defined baseline characteristics and clinical data. Overlapping charts were reviewed for accuracy. All methamaphetamine-positive cases were verified by both abstractors. Missing NIHSS or ICH scores were calculated based on available information from physical examination and imaging studies without knowledge of who was methamphetamine positive.

Methamphetamine-associated ICH was defined as any ICH patients who had a positive UDS for amphetamines or documented history of methamphetamine use by physicians and/or social workers. Analysis based on self-reported history of methamphetamine use alone would significantly underestimate the proportion of methamphetamine-associated ICH. UDS was used to identify additional methamphetamine-associated ICH. We had a standard ED Code Stroke orderset for all potential ICH patients. However, the decision to obtain the UDS was at the discretion of the on-call physicians at the time of admission. They had to check or uncheck the UDS box in the orderset for each individual patient.

The study was approved by the University of California Institutional Review Board and Ethical Standards Committee (HS#2018–4332). Informed consents were waived by IRB due to minimal risk of harm to the patients.

### Amphetamine measurements

UDS was performed using EMITII Plus Amphetamines assay (1000 ng/mL cutoff) with sensitivity and specificity of 94.3 and 93.3%, respectively [13]. Home medications were reviewed to determine potential for false positive results [14]. Patients who was taking trazodone, Adderall, bupropion, or labetalol within 2 weeks of admission were excluded from the study to minimize false postive cases [7].

### Statistical analysis

Patient were divided into groups based on the presence of a UDS for methamphetamine. Chi-squared, t-test or Wilcoxon rank-sum test analysis was used to compare the characteristics of patients with and without UDS. Univariate analysis was performed initially to assess the possible factors associated with UDS test. The cutoff value in univariate analysis for inclusion in the multivariable logistic regression was *p* <  0.1. Multivariable analysis was performed to investigate potential factors in deciding to obtain UDS after adjusting for age, sex, race, histories of smoking, methamphetamine use, anticoagulant use, hypertension, diabetes, baseline NIHSS and EVD placement. All analyses were performed using Statistical Package for the Social Sciences (SPSS) software (version 23.0). A 2-tailed value of *p* <  0.05 was considered statistically significant.

## Results

**Fig. 1:**
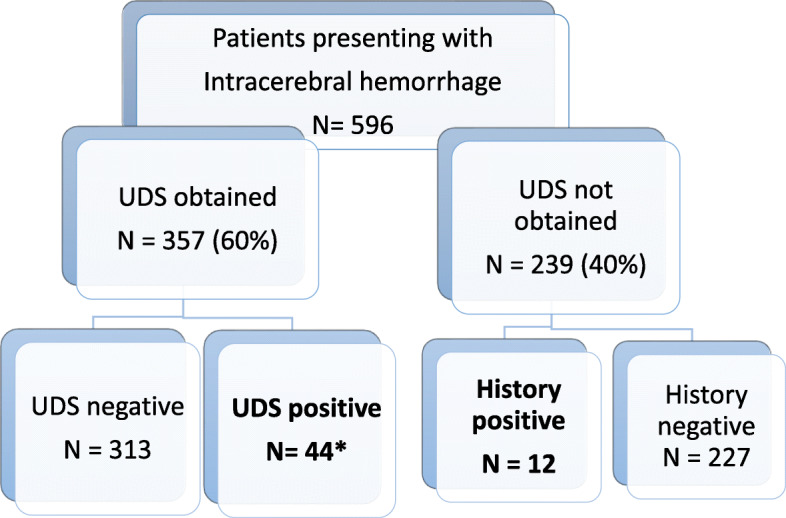
Flowsheet for identifying methamphetamine use by UDS and/or history. *19 of those with a positive UDS also reported history of methamphetamine use

Five hundred ninety-six consecutive patients hospitalized during the study period were identified to have spontaneous ICH and all were included in data analysis. As shown in Fig. 1, UDS was performed in 357 patients (60%) and positive for methamphetamines in 44 (12.3%, 44/357). In contrast, only 19 of the 357 patients with a UDS had a documented history of use (5.3%, 19/357). Clearly, anaysis based on self-reported history of methamphetamine use would significantly underestimate the Methamphetamine-associated ICH.

Among the 239 patients without UDS, 12 had history of methamphetamine abuse (5%, 12/239). Thus, a total of 56 were considered to have methamphetamine-associated ICH (9.4%). Interestingly, in both UDS and non-UDS groups, the self-reported rates of methamphatmine use were very close (5.3% vs 5%).

The demographics and clinical data of the patients with and without UDS are shown in Table 1. There were significant differences between the 2 groups. Patients screened with UDS were more likely to be younger (60 ± 16 vs. 66 ± 15, *p* <  0.001), male (62.5% vs. 50.2%, *p =* 0.003), smokers (19.4% vs. 11.8%, *p* = 0.014), with a history of methamphetamine use (8.2% vs. 0.8%, *p* <  0.001), not on anticoagulant agent (7.0% vs. 13.9%, *p* = 0.016), without diabetes (23.9% vs. 32.1%, *p* = 0.03), with higher diastolic blood pressure (181 ± 41 vs. 174 ± 37, *p* = 0.033) and systolic blood pressure (100 ± 24 vs. 91 ± 21, *p* <  0.001), having higher NIHSS scores and requiring external ventricular drain (EVD) placement. There was no significant difference in race, history of hypertension, hyperlipidemia, coronary heart disease, Glasgow Coma Scale (GCS), ICH location, IVH, ICH score or intubation.

**Table 1:** Characteristics of patients with and without UDS

Variables	UDS (***n*** = 355)	No UDS (***n*** = 237)	***p***
Age	60 ± 16	66 ± 15	**< 0.001**
Male	222 (62.5)	119 (50.2)	**0.003**
Race			
White	150 (42.3)	99 (41.8)	0.907
Hispanic	112 (31.5)	65 (27.4)	0.283
African American	13 (3.7)	15 (6.3)	0.134
Asian	80 (22.5)	60 (25.3)	0.435
Hypertension	244 (68.7)	161 (67.9)	0.837
Diabetes	85 (23.9)	76 (32.1)	**0.030**
Hyperlipidemia	66 (18.6)	52 (21.9)	0.318
Coronary Artery Disease	33 (9.3)	31 (13.1)	0.146
Anticoagulant use	25 (7.0)	33 (13.9)	**0.016**
Smoking	69 (19.4)	28 (11.8)	**0.014**
History of Meth use	29 (8.2)	2 (0.8)	**< 0.001**
Heart rate	87 ± 19	86 ± 21	0.501
Systolic blood pressure, (mmHg)	181 ± 41	174 ± 37	**0.033**
Diastolic blood pressure, (mmHg)	100 ± 24	91 ± 21	**< 0.001**
NIHSS	12 (5, 25)	11 (2, 24)	0.165
NIHSS 0–4	81 (22.8)	87 (36.7)	**< 0.001**
NIHSS 5–9	68 (19.2)	21 (8.9)	**0.001**
NIHSS > 9	206 (58.0)	129 (54.4)	0.398
GCS	13 (6, 15)	13 (6, 15)	0.899
GCS < 9	115 (32.4)	77 (32.5)	0.981
ICH location			0.654
Hypertensive^a^	212 (59.7)	136 (57.4)	
Atypical	135 (38.0)	93 (39.2)	
Multifocal	8 (2.3)	8 (3.4)	
IVH	173 (48.7)	122 (51.5)	0.513
ICH score	1 (0, 3)	2 (1, 3)	0.503
EVD	87 (24.5)	39 (16.5)	**0.019**
Intubation	158 (44.5)	101 (42.6)	0.650

Data are n (%), mean ± SD, or median (interquartile range)

^a^Hypertensive ICH locations included basal ganglia, thalamus, cerebellum or brainstem. Any other locations, including cortical or lobar hemorrahge, were considered atypical [15]

Factors that may independently influence decision to obtain UDS are explored with multivariable analysis (Table 2). Patients with a UDS were more likely to be younger than 45 (OR, 2.24; 95% CI, 1.28–3.91; *p* = 0.004), male (OR,1.65; 95% CI, 1.18–2.30; *p* = 0.003), smokers (OR,1.74; 95% CI,1.09–2.77; *p* = 0.02), have reported history of methamphetamine use (OR, 10.48; 95%CI, 2.48–44.34; *p* <  0.001), not be on anticoagulant therapy (OR,0.45;95% CI, 0.26–0.78; *p* = 0.004), not have diabetes (OR,0.68;95%CI,0.471–0.98;*p* = 0.036), have NIHSS scores > 4 (OR, 1.92;95%CI, 1.34–2.75; *p* <  0.001), or require EVD (OR,1.63;95% CI, 1.07–2.47; *p* = 0.021). Requiring EVD or intubation were included to assess if inability to obtain an oral history of drug use, and/or severity of presentation may influence decision to obtain UDS. There was no significant difference in race (*p* = 0.319). Reported history of methamphetamine use was the strongest predictor of obtaining a UDS (OR,10.48).

**Table 2:** Factors associated with obtaining a UDS in patients presenting with intracerebral hemorrhage (*n* = 596)

Variables	Odds Ratio	95% CI	***P*** Value
Age (< 45)	2.24	1.28–3.91	**0.004**
Sex (male)	1.52	1.18–2.30	**0.003**
History of meth use	10.48	2.48–44.34	**< 0.001**
History of smoking	1.74	1.09–2.77	**0.020**
History of hypertension	1.07	0.75–1.52	0.717
History of diabetes	0.68	0.47–0.98	**0.036**
Anticoagulant use	0.45	0.26–0.78	**0.004**
NIHSS > 4	1.92	1.34–2.75	**< 0.001**
Required EVD	1.63	1.07–2.47	**0.021**
Race			0.319
White	0.97	0.69–1.35	0.846
Hispanic	1.27	0.89–1.83	0.191
African American	0.56	0.26–1.21	0.136
Asian	0.88	0.60–1.28	0.496

## Discussion

In our large cohort, we demonstrate that 60% of patients presenting to our stroke center with spontaneous ICH had a UDS. UDS was positive for methamphetamine in 12.3% of these patients. By history alone, only 5.3% reported methamphetamine use. Our data indicate that analysis based on self-reported history of methamphetamine use would significantly underestimate the prevalence of methamphetamine-associated ICH. Therefore, all patients with ICH should get a UDS. Further studies are warrantied to better define methamphetamine-associated ICH.

A number of factors were identified to be associated with having a UDS. Young and male patients and individuals with history of smoking and methamphetamine use are more likely to be tested. Smokers were more likely to have a UDS, perhaps owing to the suggestion of riskier behavior. Patients with more severe presentations (requiring EVD or intubation) were more likely to be screened with UDS, possibly due to inability to give history. Reported history of methamphetamine use was the strongest predictor of obtaining UDS (OR,10.48).

Patients with a common risk factor for hemorrhage, anticoagulant therapy, were less likely to be tested for drugs. Potentially this was because a cause for hemorrhage seemed to be identified. History of hypertension was not associated with a lower chance of obtaining a UDS. As methamphetamine is a potent hypertensive agent, and surges in blood pressure may be the cause of ICH in its users [3, 4], it is essential to order UDS in patients with hypertensive ICH. Previous research has shown ethnic disparities in ordering drug screens in patient with ICH, with young African Americans being screened more often [16]. In our study, we found no significant racial bias in obtaining UDS for patients with spontaneous ICH.

Of note, we excluded patients with documented recent use of trazodone, Adderall, bupropion, or labetalol to minimize the false positive rates [7]. However, due to lack of information on other medications that may potentially cause false-positive results [14], we were unable to calculate the true false positive rate. Amphetamine is detectable in the urine for 2–3 days after ingestion. We had no detailed information on recent versus remote use of methamphetamine to calculate false negative rate.

The EMITII Plus Methamphetamine assay used for UDS at our medical center also detects barbiturates, cocaine, benzodiazepine, methadone, opiates, phencyclidine, alcohol, THC, propoxyphene, MDMA [13]. Of all the substances, only methamphetamine and cocaine are significant risk factors for ICH. In our study cohort, only 2 patients were also tested positive for cocaine [7]. Other stimulants therefore were unlikely a significant confounding factor for our ICH study.

Previous studies have indicated the importance of evaluating young patients presenting with ICH for drug use [4, 11]. There was a bias in ordering UDS more often for younger, male patients in this study. Nowadays patients presenting to the emergency department (ED) with methamphetamine-related complaints are of a wide age range with 18% older than 45 [17]. ED visits for methamphetamine associated complaints are increasing nationwide, particularly in the Pacific Rim [5, 6, 8–10],

Results from our single center study indicate that a significant number of ICH patients were not getting a UDS. Such practice may limit our ability to fully understand the extent of this drug epidemic and its association with ICH. Although the advancement of neurocritical care has improved survival for patients with ICH [18], prevention may be the best strategy for methamphetamine-associated ICH. The optimal interventions to address drug abuse are beyond the scope of this paper, however this study serves to increase cognizance about the potential underestimation of methamphetamine-associated ICH.

After identifying the disparity in ordering UDS at our stroke center, we have eliminated the check box in our orderset to make UDS the default test for all potential ICH patients.

Interestingly, a recent ICH study using the 2004–2014 National Inpatient Sample showed significant rural-urban disparities in drug abuse and ICH mortality [19]. ICH patients hospitalized in rural hospitals were found to have a lower drug abuse rate (1.2% vs 3.8%, *p* < 0.001) but two times the odds of dying (OR 2.07, 95% CI 1.77–2.41, *p* < 0.001) compared to those in urban hospitals. In our previous study [7], there was no significant difference in mortality between methamphetamine-associated versus non-methamphetamine ICH (18.0% vs. 24.6%, *p* = 0.267). We did not look into the rural-urban disparities in methamphetamine use and associated ICH in our single center study. The prevalence of drug abuse in our ICH patient population appeared to be much higher than that reported in the National Inpatient Sample study.

There are limitations to this study. First, this is a single-center retrospective study with limited generalizability. Second, in this retrospective study, it is not possible to quantify the amount, route, frequency and duration of the methamphetamine abused [7]. Some patients may have had a remote history of methamphetamine use; it is unclear how much their remote use predisposed them to hypertension and hemorrhage. Other patients might be occasional or new users of methamphetamine, and the drug abuse may have not contributed to the ICH. Third, there was also no reliable test to differentiate methamphetamine-associated ICH from spontaneous hypertensive ICH. Lastly, complete information on patient’s home medications, recent vs remote use of methamphetamine, other drug use or psychiatric history were not availabe for evaluating false positive or false negative rate of UDS test and other confounding factors.

## Conclusions

In our study cohort, UDS identified 12.3% of ICH patients with methamphetamine use as compared to 5.3% per documented history of drug use. UDS was more often ordered in younger, male, and patients with history of smoking and methamphetamine use. There was no racial bias in ordering UDS. UDS should be considered in all patients with ICH to better define methamphetamine-associated ICH.

## Data Availability

The datasets used and/or analyzed during the current study are available from the corresponding author on request.

## Abbreviations

ED: Emergency department
EVD: External ventricular drain
GCS: Glasgow Coma Scale
ICH: Intracerebral hemorrhage
ICU: Intensive care unit
IVH: Intraventricular hemorrhage
LOS: Length of stay
NIHSS: National Institutes of Health Stroke Scale
UDS: Urine drug screen
